# Supplementary material for: An analysis of retracted papers in Computer Science
Source: PLoS One. 2023 May 9;18(5):e0285383. doi: 10.1371/journal.pone.0285383 (PMC10168558; doi:10.1371/journal.pone.0285383)
Supplement: Attachment — Submitted filename: PLOS-One-ResponseToReviewers.pdf [file pone.0285383.s001.pdf]

**id**	**done?**	**text**	**response**
JR1		Please ensure that your manuscript meets PLOS ONE's style requirements, including those for file naming. The PLOS ONE style templates can be found at;<br>https://journals.plos.org/plosone/s/file?id=wjVg/PLOSOne_formatting_sample_main_body.pdf and<br>https://journals.plos.org/plosone/s/file?id=ba62/PLOSOne_formatting_sample_title_authors_affiliations.pdf	Fig. -> Fig<br>Figure files should be saved as “Fig1.tif”, “Fig2.eps”, etc.<br>Acceptable file formats for figures are “.tif”, “.tiff”, and “.eps”<br>Since LaTeX can't directly render .tif files we continue to use png but will also supply tif files separately.
JR2		We note that you have provided additional information within the Acknowledgements Section that is not currently declared in your Funding Statement. Please note that funding information should not appear in the Acknowledgments section or other areas of your manuscript.<br>We will only publish funding information present in the Funding Statement section of the online submission form.<br>Please remove any funding-related text from the manuscript and let us know how you would like to update your Funding Statement. Currently, your Funding Statement reads as follows: “The author(s) received no specific funding for this work.” Please include your amended statements within your cover letter; we will change the online submission form on your behalf.	We have attempted on repeated occasions to explain the situation to PLoS ONE staff and explore what they want. Eventually we received a message from a PLoS editor saying that the enquiry has been forwarded to a senior staff member but we've not had a reply despite reminders. By contrast, the academic journal editor has been **very helpful** and his proposal is to go with our suggestions.<br><br>Basically, I'm the recipient of a research award from the Swedish Research Council which pays for my dual appointment at Gothenburg University. It isn't to do any particular research but this paper describes some work I undertook whilst in receipt of their general funding. I thought it courtesy to mention this. We've updated the acknowledgements to state:<br>"Martin Shepperd was supported by the Swedish Research Council as the recipient of the 2022 Tage Erlander research professorship and by a sabbatical from Brunel University London**, although not specifically for this research**. ..."
JR3		In your Data Availability statement, you have not specified where the minimal data set underlying the results described in your manuscript can be found. PLOS defines a study's minimal data set as the underlying data used to reach the conclusions drawn in the manuscript and any additional data required to replicate the reported study findings in their entirety. All PLOS journals require that the minimal data set be made fully available. For more information about our data policy, please see http://journals.plos.org/plosone/s/data-availability.<br>Upon re-submitting your revised manuscript, please upload your study’s minimal underlying data set as either Supporting Information files or to a stable, public repository and include the relevant URLs, DOIs, or accession numbers within your revised cover letter. For a list of acceptable repositories, please see http://journals.plos.org/plosone/s/data-availability#loc-recommended-repositories. Any potentially identifying patient information must be fully anonymized.<br>Important: If there are ethical or legal restrictions to sharing your data publicly, please explain these restrictions in detail. Please see our guidelines for more information on what we consider unacceptable restrictions to publicly sharing data: http://journals.plos.org/plosone/s/data-availability#loc-unacceptable-data-access-restrictions. Note that it is not acceptable for the authors to be the sole named individuals responsible for ensuring data access.<br>[illegible]	Again, we have tried to reach out to the professional staff at PLOS ONe without success. Again the academic editor has been **most helpful** and his suggestion is to submit following our suggestion below.<br><br>As we have explained "we are unable to make the Retraction Watch Database available due to a data use agreement that prohibits publishing more than 2% of the data set. This requirement arises because, in order to fund Retraction Watch’s continued operations, given that their initial grants have ended, they are licensing their data to commercial entities. Therefore researchers will need to approach Retraction Watch directly to obtain the same data set." (lines 417-424).<br><br>We share our code on zenodo but anyone wishing to reproduce the work must approach RetractionWatch directly. They say they will not withhold permission from any bona fide researcher but we can't do this directly.
JR4	Y	Please review your reference list to ensure that it is complete and correct. If you have cited papers that have been retracted, please include the rationale for doing so in the manuscript text, or remove these references and replace them with relevant current references. Any changes to the reference list should be mentioned in the rebuttal letter that accompanies your revised manuscript. If you need to cite a retracted article, indicate the article’s retracted status in the References list and also include a citation and full reference for the retraction notice.	To the best of our knowledge the reference list is correct. We have decided not to cite retracted papers as explained in FN4 (p4).
R1.1	Y	I have just one main suggestion: considering the fact that in some cases retraction watch classifies the retracted papers under more than just one subject. e.g., a retracted paper in CS might be classified under Medicine as well. I think discussing a little bit more this fact, or even present some stats regarding the subjects that appeared the most together with CS will enrich the overall findings and discussion.	Thanks for this interesting idea. We have computed the number of subject areas per paper, added a histogram plus 2 new paragraphs of text on p6 (RQ1).

R1.2	Y	Please cite the document defining the reasons of retraction you have listed: "… "A non-exhaustive list includes: …"	We've added the source i.e., RetractionWatch and indicated there are a total of 102 reasons.
R1.3	Y	I like the discussion regarding the reason of citation (or citation function), you might consider including other relevant articles, such as: (1) "Teufel, S., Siddharthan, A., & Tidhar, D. (2006). Automatic classification of citation function. Proceedings of the 2006 Conference on Empirical Methods in Natural Language Processing - EMNLP '06, 103. https://doi.org/10.3115/1610075.1610091", (2) "Heibi, I., & Peroni, S. (2022). A protocol to gather, characterize and analyze incoming citations of retracted articles. PLOS ONE, 17(7), e0270872. https://doi.org/10.1371/journal.pone.0270872" to classify the citation reason. (3) "Tuarob, S., Kang, S. W., Wettayakorn, P., Pornprasit, C., Sachati, T., Hassan, S.-U., & Haddawy, P. (2020). Automatic Classification of Algorithm Citation Functions in Scientific Literature. IEEE Transactions on Knowledge and Data Engineering, 32(10), 1881–1896. https://doi.org/10.1109/TKDE.2019.2913376"	Thanks for the references. We have added: "Interestingly a number of researchers have sought to automate the process of determining citation purpose using a range of contextual data along with linguistic cues, e.g., Teufel et al. [20], more recently Heibi and Peroni [21], and in the specific domain of algorithm citation Tuarob et al. [22]. "
R1.4	Y	while discussing the "article type", I think you should talk a little bit more (and refer to relevant previous studies) about what is the most frequent publishing typology in Computer Science. This will let us have the right perception regarding the numbers of retraction for each different typology in CS.	We have extended the paragraph that discuss this to:<br>"Next, we consider article type. Unfortunately, the database contains 80 distinct types of articles some of which seem to overlap and others are not relevant for Computer Science such as Clinical Study. Specifically, there are 19 different types of paper for CS (the details are given in Table~\ref{Tab:ArtType}. However, there are some clear patterns. The largest category is Conference Abstract/Paper for which there are 1947 retractions, followed by 727 Research Articles. Some 19 papers are classified as Reviews or Meta-analyses (which is slightly fewer than the 28 we detected by searching in the title for either `review' or `meta-analysis'). The picture is somewhat complicated by the fact that is another multi-valued attribute with some categories being composites such as ``Article in Press; Research Article''. "<br>In addition we have added a new table of frequency counts. Ultimately the analysis is somewhat restricted by the use of composite categories.
R1.5	Y	You have mentioned that CS has a high number of retractions (65.3%) reporting "Little or no information available" compared to the general trend (27.4%). This fact itself could be considered for a whole new study, yet, can you discuss it more (based on your experience and impressions) in the conclusions (or as part of a "Further work") .	Thanks for the suggestion.<br><br>We append: "Speculating, one possible reason is the prevalence of conferences in CS and the risk that the devolving of editorial and refereeing responsibilities to local groups could leave publishing vulnerable to exploitation and refereeing cartels. In such situations publishers undertake bulk retractions and possibly make no distinction between papers leading to bland and uninformative retraction notices." to the discussion in RQ1.<br><br>And under further work we add a new paragraph: "Another theme that would benefit from further investigation is the low proportion of meaningful retraction notices in CS compared to other disciplines. It is clear that meaningful reasons for retraction would be of value to the community and potentially to the authors especially when the reasons fall into the category of `honest error'. Although we speculate that one driver could be the many conferences and subsequent bulk retractions it would clearly

R1.6	Y	“… we noticed that the paper ”Obstacle Avoidance Algorithms: A Review” has …” Although you talk about a retracted paper, citing it is not prohibited, as long as it is clearly stated the fact that the article is retracted in both the text and its reference entry (as it has been suggested also by Retraction Watch, see https://retractionwatch.com/2018/01/05/ask-retraction-watch-ok-cite-retracted-paper/#:~:text=It's%20perfectly%20fine%20to%20cite,retracted%20papers%20in%20our%20database.). This is still of course a personal decision, yet, I think citing a retracted article is completely fine, the more delicate aspect is regarding “how it is cited“ (of course in a world where citations treated only from a quantitative point of view are used to rank authors and journals, this fact is very delicate and indeed a subject of future discussion). You have other similar situations in the rest of your paper.	Thanks for this suggestion. We feel there's an ambiguity when citing even a clearly retracted paper and for this reason we prefer not to add a formal citation. However, we add a footnote to make our reasoning clearer.
R1.7	Y	“However, Bar-Ilan and Halevi [13] found that negative citations are rare and do not well predict retracted papers. … whatever the context, high levels of citations to retracted papers are a considerable cause for concern.” the work you are citing here analyzed a specific use case, so be careful in generalizing their findings to other domains and case study, such as the one you are analyzing (CS). Your following statement is very strong, I suggest you to reword this sentence in a “supposition“ style.	Thanks. We re-word as "However, Bar-Ilan and Halevi~\cite{BarI17} found in their analysis of 238 citations to the 15 most cited but retracted papers (1995-2014) from the Elsevier ScienceDirect database that negative citations are rare and do not well predict retracted papers. Of course a caveat is that 15 papers is a small sample. Ultimately we believe that whilst every retracted paper and its citations are in some sense its own story, we still strongly believe that, whatever the context, high levels of citations to retracted papers are a considerable cause for concern.".
R2.1	Y	On page 2, the US Office for Research Integrity’s definition for research misconduct was stated but no intext citation was given and the appropriate document from US Office for Research Integrity was not included in the list of references.	Reference added.
R2.2	N	Instead of subsuming the methodology used in addressing each research question under the section on Analysis and Results, the authors should add a section on methodology, where methods used in extracting data from Retraction Watch as well as the methods used in analyzing the collected data are described.	This is an interesting point. Ordinarily we would agree that your suggested structure is more commonplace and therefore easier for the reader. However, in this case our methods are trivial in that we simply use the RetractionWatch database. Beyond that, the details are in the RMarkdown file (ie our code) which we share. We feel flow and readability is better if we simply move straight on to our analysis and discussion. But if the editor feels otherwise of course we will reorganise the paper.
R2.3	Y	On page 13, recommendation #4 needs a little bit of explanation.	Thanks. We've rephrased the first sentence as "the active adoption of tool support to help researchers better identify retracted papers e.g., the welcome integration of the RW database into the Zotero bibliographic management tool."
R2.4	Y	The manuscript lacks clarity in a few areas and contains a few typographical and grammatical errors. It would therefore benefit from some editing.	We have carefully proofread the paper, made a few corrections and tried to improve the clarity.
General		While revising your submission, please upload your figure files to the Preflight Analysis and Conversion Engine (PACE) digital diagnostic tool, https://pacev2.apexcovantage.com/. PACE helps ensure that figures meet PLOS requirements. To use PACE, you must first register as a user. Registration is free. Then, login and navigate to the UPLOAD tab, where you will find detailed instructions on how to use the tool. If you encounter any issues or have any questions when using PACE, please email PLOS at figures@plos.org. Please note that Supporting [illegible]	
